# Primary central nervous system angiitis: a case report

**DOI:** 10.1097/MS9.0000000000002205

**Published:** 2024-05-27

**Authors:** Zahraa M. M. Zeer, Yasmin N. A. Arda, Hasan Abu Salim, Mohammad M. Abbas

**Affiliations:** a Faculty of Medicine, Al-Quds University, Jerusalem; b Jenin Governmental Hospital, Jenin, Palestine

**Keywords:** angiitis, case report, histology, misdiagnosis, nervous system

## Abstract

**Introduction:**

Primary central nervous system angiitis is a rare idiopathic vasculitis that is limited to the central nervous system. It has a wide range of clinical presentations that can mimic other vasculopathies.

**Case presentation:**

A 24-year-old female patient presents with various non-specific neurological complaints in a progressive course. After a challenging diagnostic work-up, she was diagnosed by tissue biopsy to have primary central nervous system angiitis.

**Discussion:**

Although primary central nervous system angiitis has been reported increasingly recently, its pathogenesis is still unknown, and its diagnosis is still very challenging. No universal criteria have been adopted, and there is no laboratory test or imaging modality with sufficient sensitivity and specificity to confirm the diagnosis and exclude other mimickers.

**Conclusion:**

To prevent misdiagnosis, clinicians treating patients with suspected primary central nervous system angiitis should be aware of its differentials.

## Introduction

Highlights

- To prevent misdiagnosis, clinicians treating patients with suspected primary central nervous system angiitis should be aware of its differentials.
- No laboratory test or imaging modality with sufficient sensitivity and specificity to confirm the diagnosis of primary central nervous system angiitis.
- Differentiating primary CNS angiitis from other mimickers is very crucial as early treatment with immunosuppressive therapy is considered a good prognostic factor.

Primary central nervous system (CNS) angiitis is an idiopathic exclusive inflammation of the arteries and veins of the CNS and its dural reflections, especially the small and medium cerebral vessels^1^. It has a wide range of clinical manifestations with headache and encephalopathy accompanied by multifocal neurological symptoms being the most common presentations^2^. It has an annual incidence rate of 2.4 per one million people. Men are more affected than women with an average age of presentation of 50 years^3^. Other than the heterogenous clinical presentations, accurate diagnosis of this condition poses some challenges as there are no laboratory tests or imaging modalities with sufficient sensitivity and specificity to confirm the diagnosis and exclude all mimickers of vasculitis. A definitive diagnosis is given by biopsy. Histologically, this disease has three patterns: granulomatous, lymphocytic, and necrotized in order of frequency^4^. Differentiating primary CNS angiitis from other mimickers clinically and radiologically is very crucial as early treatment with immunosuppressive therapy is considered a good prognostic factor^5^. In addition, other mimickers of primary CNS angiitis may be exacerbated by immunosuppressive therapy^6^. In this case report, we present a case of a 24-year-old female patient who presents with various neurological complaints in a progressive course. After a challenging diagnostic work-up, she was diagnosed to have primary CNS angiitis. This case report has been reported in line with the SCARE Criteria^7^.

### Case presentation

**Figure 1:**
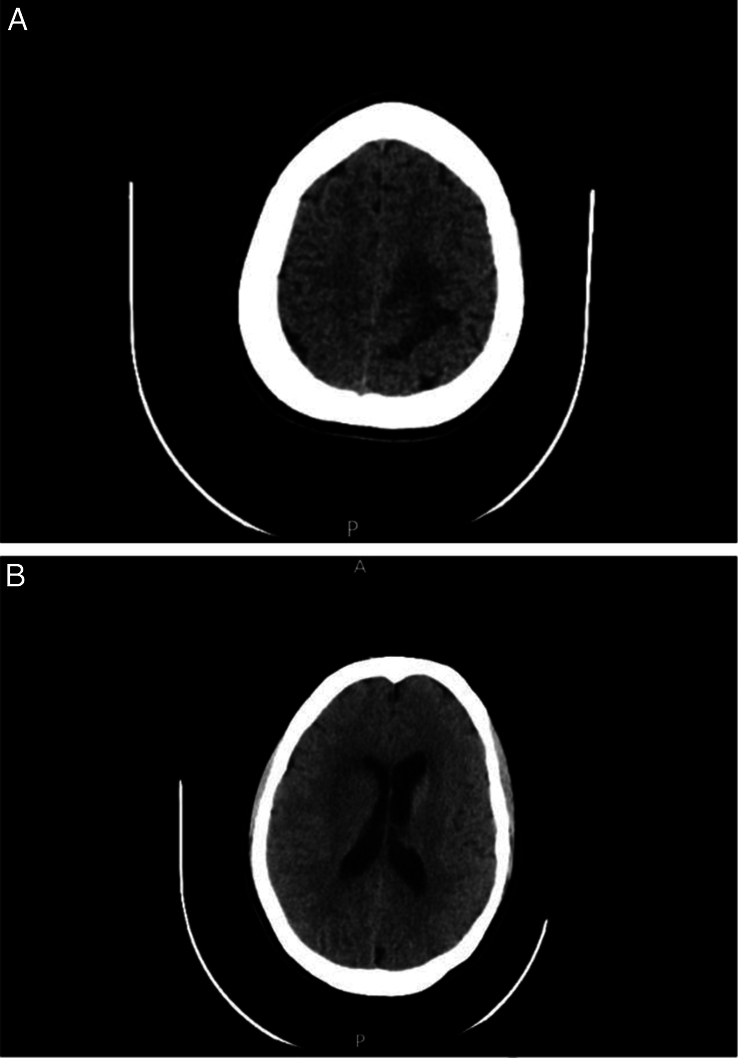
Cranial computed tomography shows few areas of brain parenchymal oedema in the left para-falci area (A) and the right periventricular area (B) with no intracranial haemorrhage, midline shift or bone lesions.

A 24-year-old woman presented complaining of acute changes of behaviour to the extent that she could not recognize her family. Her past medical history revealed persistent progressive headache for 2 months. Her headache was bifrontal and tension-like in nature; it persisted throughout the day and night but with higher intensity in the mornings. The headache partially improved with analgesia, and it was associated with blurred vision, nausea, and sometimes with vomiting. She described her headache’s severity as 7 out of 10. The patient denied a history of skin rash, night sweats, joint pain, changes in appetite, or weight loss. On admission, the patient was confused. On neurological examination, she had an ataxic gait to the left side, amnesia to recent events, and numbness involving her right upper and lower limbs. The power was 5/5 in all extremities. The knee reflex was greater at the left side. Cranial nerves were intact. There was no evidence of papilledema after the ophthalmological examination. Cranial computed tomography (CT) showed few areas of brain parenchymal oedema in the left para-falci area and in the right periventricular area with no intracranial haemorrhage, midline shift, or bone lesions (Fig. 1). Her white blood cell count was 15 000 per microliter, erythrocyte sedimentation rate (ESR): 30 mm/h, C-reactive protein (CRP): 12.2 mg/dl. Otherwise, routine laboratory results were unremarkable. Hepatitis Bs antigen and hepatitis C virus antibody tests were negative. Immunological work-up, which included ANA, ENA, anti-double strand DNA antibody, anti-cardiolipin IgM and IgG, anti-beta-2-glycoprotein I IgG and IgM, ANCA, anti-aquaporin 4, anti-MOG antibodies and anti-phospholipid screen were all negative.

**Figure 2:**
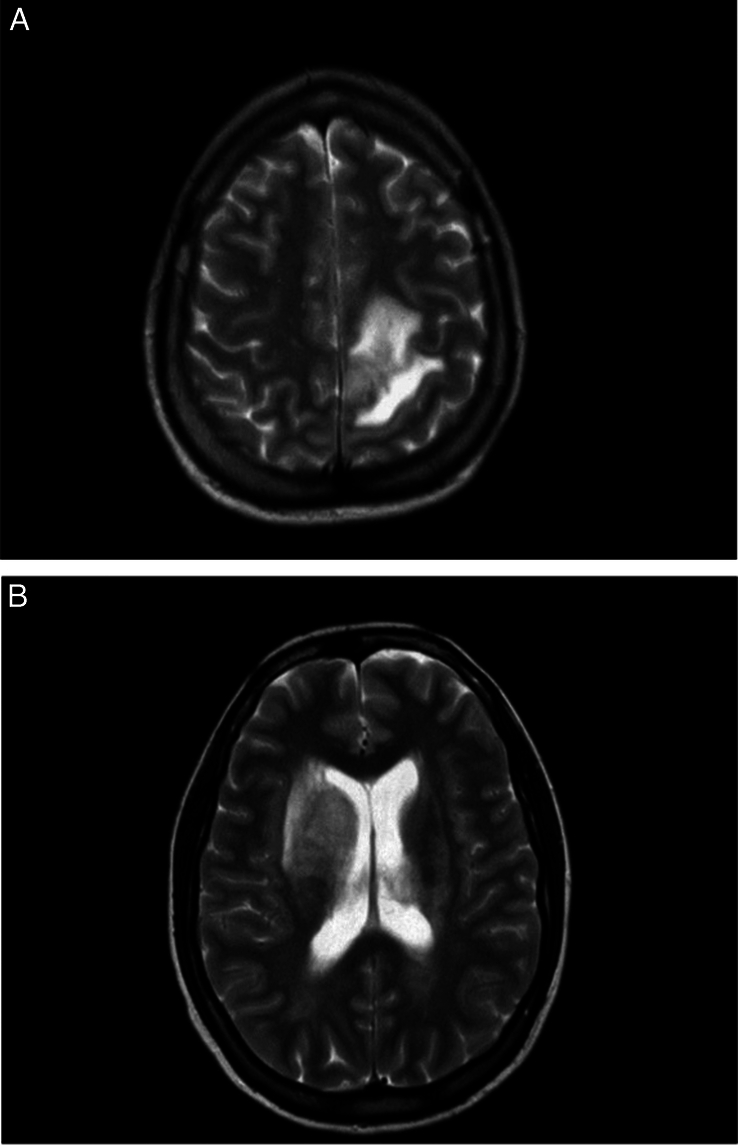
MRI shows multiple bilateral periventricular hyperintensity lesions on T2/FLAIR mainly in the right basal ganglia, both caudate nuclei, left thalamus, and left para-falci grey matter. They were surrounded by white matter oedema. Serpiginous vascular structures were seen after IV contrast gadolinium administration. Fluid-attenuated inversion recovery (FLAIR).

Brain MRI showed multiple bilateral periventricular hyperintensity lesions on T2/FLAIR mainly in the right basal ganglia, both caudate nuclei, left thalamus, and left para-falci grey matter. They were surrounded by white matter oedema. Serpiginous vascular structures were seen after IV contrast gadolinium administration (Fig. 2). Additionally, MRA and MRV were unremarkable.

During her hospital stay, the patient received 60 g of mannitol and metoclopramide 10 mg twice daily if needed, and her behavioural changes improved. The patient was discharged after stabilization as her brain biopsy planning was pending.

**Figure 3:**
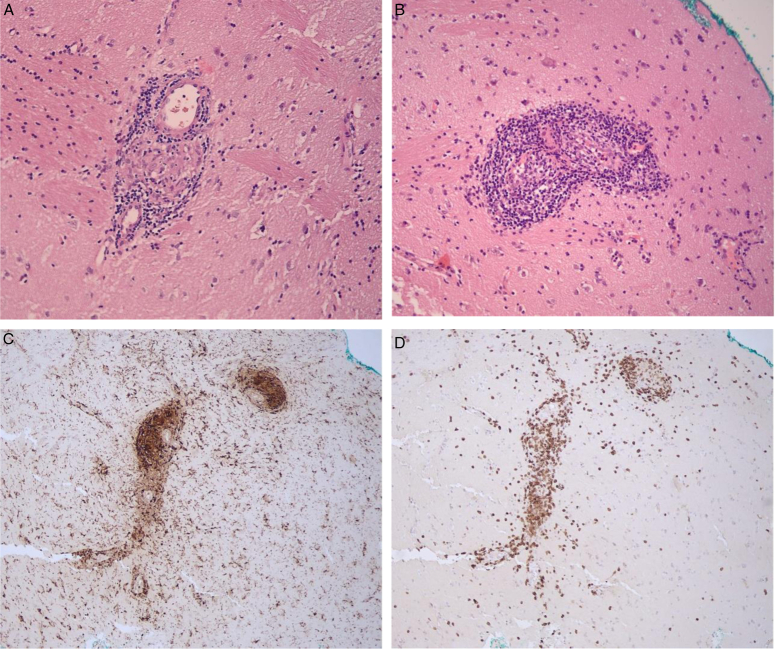
Pathological changes in primary central nervous system angiitis. (A). (hematoxylin and eosin, 40×) perivascular lymphoid infiltration in the brain with small non-necrotizing granuloma. (B) (hematoxylin and eosin, 100×) Dense lymphoid perivascular infiltration with occlusion of the lumen of arteriole. (C) (immunoperoxidase, 100×) Immunohistochemical staining for CD68 (marker for macrophages), showing perivascular infiltration by macrophages. (D) (immunoperoxidase, 100×) Perivascular infiltration by T-lymphocytes, positive for CD3.

A month later, the patient was re-admitted with deterioration in the form of disorientation, headache, recurrent vomiting, difficulty in speech, recurrent convulsions, and weakness involving the right side of her body with bilateral abducent nerve palsy. CT was done again and showed the same findings as before. A brain biopsy was performed and showed small blood vessels with lymphocytic infiltration, endothelial proliferation, and granulomas (Fig. 3). Table 1 shows all the tests which were done during the patient’s work-up with their results.

**Table 1:** work up tests and their results.

Work-up tests	Result
White blood cells (WBC)	15 000 per microliter
ESR	30 mm/h
CRP	12.2 mg/dl
Hepatitis Bs antigen and hepatitis C virus antibody	Negative
Immunological work-up: ANA, ENA, anti-double strand DNA antibody, anti-cardiolipin IgM and IgG, anti-beta-2-glycoprotein I IgG and IgM, ANCA, anti-aquaporin 4, anti-MOG antibodies and anti-phospholipid screen were all negative.	Negative
Brain MRI	Multiple bilateral periventricular hyperintensity lesions on T2/FLAIR mainly in the right basal ganglia, both caudate nuclei, left thalamus, and left para-falci grey matter. They were surrounded by white matter oedema. Serpiginous vascular structures were seen after IV contrast gadolinium administration
MRA and MRV	Unremarkable
Brain biopsy	Small blood vessels with lymphocytic infiltration, endothelial proliferation, and granulomas

ANA, antinuclear antibody; ANCA, anti-neutrophilic cytoplasmic antibody; CRP, C-reactive protein; ENA, extractable nuclear antigen; ESR, erythrocyte sedimentation rate; MOG, myelin oligodendrocyte glycoprotein; MRA, Magnetic resonance angiography; MRV, magnetic resonance venography.

Primary CNS angiitis was diagnosed after excluding other differential diagnoses, including systemic vasculitis, CNS lymphoma and other malignancies. Following her diagnosis, the patient was started on methylprednisolone 1 g IV once for 5 days. Rituximab was added later to her regimen due to her complaint of frequent headaches; the patient received rituximab 1 g IV followed by a second dose 14 days later. She is currently scheduled to receive rituximab every 6 months. She also receives 100 000 units of vitamin D injection every 2 weeks. During follow-up, the patient demonstrated significant clinical improvement in her symptoms. Since starting rituximab, the patient had one relapse during which she complained of numbness in her upper and lower limbs. She also complained of dizziness, double vision, black spots and right lower limb weakness with minimal improvement on methylprednisolone. She was referred for urgent plasmapheresis after which her symptoms rapidly improved. Since then, the patient has been doing well with minimal symptoms of occasional headaches.

## Discussion

### Definition

Primary angiitis of the central nervous system (PACNS) is a rare and poorly understood type of vasculitis. It is considered one of the vasculopathies that pose a real diagnostic challenge for physicians^8^. It is limited to the parenchyma of the brain and spinal cord, arteries, and meningeal veins that leads to inflammation and destruction of the vessels, especially the small-medium vessels^3^. This leads to narrowing or occlusion of these blood vessels^9^. Subsequently, haemorrhagic and ischaemic events will occur in the adjacent tissues, which will cause diverse clinical presentations^6^. It constitutes about 1.2% from all the vasculitis that involve the CNS^3^. It was first described by Cravioto and Feigin in mid 1950s^8^. In 1988, Calabrese and Mallek suggested clinical diagnostic criteria for this entity which includes (1) the presence of unexplained neurological manifestations after doing clinical evaluation and complete laboratory work-up, (2) evidence of arteritis in angiography and/ or biopsy, (3) excluding systemic vasculitis or any secondary conditions that may cause the previous described angiography or biopsy findings^10^. Although these criteria seem to be straightforward, the second and third criteria remain challenging as we will discuss further^9^.

The exact pathogenesis is still unknown. A Th1-mediated response is thought to cause the granulomatous nature of the vascular inflammatory lesions^3^. What triggers this immune mediated cascade and its propagation that leads to blood vessel destruction is still unknown^11^.

### Epidemiology

The annual incidence of this disease is reported to be 2.4 per 100 000 people. Most reported cases are from Europe and North America^3^. It can occur at any age, but it occurs predominantly in the fourth to sixth decades^2^. The disease shows male predilection with a 2:1 male-to-female ratio among affected patients^12^.

### Clinical presentation

Patients with PACNS present with different courses ranging from hyperacute to chronic, with diverse clinical features and multiple patterns and outcomes^4^. In most cases, disease onset is insidious with slowly progressive symptoms. This is one of the causes that lead to delay between the onset of symptoms and diagnosis. Salvarani and colleagues conducted an analysis of 101 cases and found that the average time from symptom onset to diagnosis was slightly over 5 weeks^9^. On the other hand, acute presentations like transient ischaemic attack or stroke occur in 30–50% of the patients^13^.

Headache is reported as the most common initial symptom (63%) followed by cognitive impairment (50%). Later in the disease course, focal symptoms may appear, which include: hemiparesis (44%), stroke (40%), aphasia (28%), transient ischaemic attack (28%), ataxia (19%), seizures (16%), dysarthria (15%) and blurred vision or decreased visual acuity (11%). In less than 10% of cases, less common manifestations may occur, such as amnesic syndrome and spinal cord manifestations like paraparesis, quadriparesis, parkinsonism, vertigo, dizziness or cranial nerve palsy^3^.

The patterns and disease outcomes are also reported to be heterogenous such as fulminant-disease onset, spinal involvement, prominent leptomeningeal enhancement, and negative cerebral angiography^4^.

### Diagnosis

Diagnosis of PACNS still has many challenges despite being increasingly reported in the past years. This attributes to the rarity of this entity, which presents with a wide range of presentations^13^. In addition, there is no laboratory test that has sufficient specificity and sensitivity to confirm the diagnosis and exclude other mimickers^4^.

For patients suspected to have PACNS, MRI is the modality of choice. It is abnormal in 90%-100% of patients with PACNS^5^. Typical findings which are seen are non-specific which include in order of frequency discrete or diffuse supra‐ and infratentorial lesions most commonly involving the superficial white matter, followed by the deep grey matter, the deep white matter, and the cerebral cortex^14^. Fifty percent of the patients are noted to have ischaemic lesions. On the other hand, haemorrhagic lesions are seen in 10–14% of the patients with intracerebral haemorrhage being the most common type 57–60%. Infarction lesions are more common to be bilateral and supratentorial but single arterial territory infarcts and infratentorial lesions are also reported. The presence of microbleeds and leptomeningeal enhancement also suggest the diagnosis of PACNS^11^. Mass lesion is reported in 15% of the cases^8^. CSF examination in patients with PACNS is abnormal in 80–90% of the patients and shows elevated proteins and lymphocyte pleocytosis^2^. CSF analysis is mainly ordered to rule out other infectious and neoplastic causes^11^. In general, if the patient has combined normal results in MRI and CSF analysis, PACNS is unlikely as this combination has a high negative predictive value^3^.

Cerebral angiography is considered the radiological gold standard investigation for PACNS. Medium-sized vessels show multifocal beading with alternating between stenoses and dilatation. On the other hand, angiography may be normal, especially if the vasculitis is limited to small blood vessels. In biopsy-proven cases, estimated average sensitivity and specificity are 60%, 30%, respectively^13^. Its low specificity is attributed to the multiple mimickers that show the same presentation as PACNS on angiography like atherosclerosis, radiation vasculopathy, fibromuscular dysplasia, and reversible vasoconstriction syndrome^5^. These significant variations in sensitivity and specificity pose a real challenge on relying on imaging alone to confirm or exclude the diagnosis^13^.

In view of the above, biopsy and histopathology is considered the gold standard for the diagnosis. PACNS has three categories histologically: granulomatous, lymphocytic and necrotizing. The most common type is granulomatous which shows vasculocentric mononuclear and granulomatous inflammation (50–60%). Lymphocytic angiitis is the second most common type which shows prominent lymphocytic inflammation associated with plasma cell infiltration and destruction of vessels (25%). Necrotizing angiitis which is associated with transmural fibrinoid necrosis is considered the least common type (14–22%). Intracranial haemorrhage, vessel rupture and aneurysmal dilatation may happen after vessel wall thickening due to fibrinoid necrosis. In some patients, multiple histological features may coexist^1,15^.

Although biopsy and histological confirmation is considered the standard for confirming the diagnosis, this procedure has many limitations. It is a highly invasive procedure that requires the skills of an experienced neurosurgeon. Special stains and cultures should be performed even in the presence of vasculitis to exclude secondary causes of vasculitis like occult infection^3^. In addition, false negative results are reported to be about 35% as this disease is considered highly patchy and has a segmental nature^2^. In order to reduce the possibility of false negative results, biopsy should be targeted to the radiologically abnormal tissue and should be sampled from both cortical and meningeal tissues^13^. If the lesion is not accessible or if it is located in the eloquent cortex, then a blind biopsy could be done from areas that have lower risk of causing neurological deficits like the non-dominant frontal lobe or the temporal pole^11^.

Frequent discrepancy between angiography and pathology results in the diagnosis of PACNS was demonstrated by McVerry and colleagues systematic review. Biopsies were found to be twice as likely to be normal than abnormally in patients with abnormal angiography, and this reveals the high risk of false positive results when relying on angiography alone. On the other hand, in cases with classic histopathology, angiography was found to be three times more likely to be normal than abnormal. This may highlight the limitation of angiography in visualizing vascular changes in medium to small blood vessels^12^. As a result, Birnbaum and Hellmann proposed some changes to the previous criteria that was established by Calabrese and Mallek in 1988^16^. These changes highlight the importance of histopathological findings as the gold standard for diagnosis rather than considering is as equivalent to angiographic findings^12^, which means to consider a definite diagnosis if the biopsy result confirms the presence of vasculitis. In addition, in cases that have no tissue confirmation, a probable diagnosis given for a patient with a high probability of abnormal findings on an angiogram combined with CSF profile consistent with PACNS and abnormal finding on MRI^16^.

In order to establish the diagnosis of PACNS, secondary causes of vasculitis should be excluded. These causes are various and could be categorized into infectious and non-infectious. Infectious causes include bacterial, viral, fungal and parasitic organisms. Bacterial causes include Treponema pallidum, Mycobacterium tuberculosis, Borrelia burgdorferi, Rickettsia species, and Bartonella henselae. Varicella Zoster, HIV, Hepatitis C, Parvovirus B19, and cytomegalovirus are considered the key causative viruses. The most common non-infectious causes include systemic vasculitis, connective tissue disorders, malignancies (lymphoma, lymphoid granulomatosis, paraneoplastic), drug exposure (cocaine and amphetamine), and other autoimmune diseases^8^. Systemic vasculitis is considered one of the most common differential diagnoses; it presents with constitutional symptoms and serological markers that indicates systemic inflammation^4^.

Other important differential diagnoses include Abeta-related angiitis (ABRA), which may complicate cerebral amyloid angiopathy; however, this type of angiitis has a higher age of presentation than PACNS^2^.

In addition, reversible vasoconstriction syndrome (RVCS) is one of the differentials that should be ruled out. PACNS and RVCS may have the same presentation, and both may cause cerebral infarction or haemorrhage. However, sudden and intense thunderclap headache suggests RVCS^10^. 11–12% of patients with PCNSA present with intracerebral haemorrhage followed by subarachnoid haemorrhage. In patients with RCVS, subarachnoid haemorrhage is more common that parenchymal haemorrhage^15^. In addition, RVCS shows multifocal areas of reversible vasoconstriction on angiography, which may be worse than the clinical presentation^13^. To confirm the diagnosis of RVCS these reversible vasoconstrictions resolve on repeat imaging within 12 weeks of the presentation^15^. RVCS as one of the notable mimickers should be excluded as using corticosteroid can be deleterious and considered as an independent factor for a worse prognosis^11,10^.

The diagnosis of our presented case faced several challenges till the final diagnosis was revealed. First, the patient complained of diverse symptoms, which broadened our differential diagnosis. Additionally, the patient waited for a month before undergoing the brain biopsy due to the lack of facilities to perform it in our hospital, and we could not start her on steroids or other medications during this time to avoid interfering with the pathology results.

### Treatment

All information on treatment is based on retrospective observational data and clinical experience as there are no randomized clinical trials or prospective studies to guide therapy^4^. Corticosteroids, immunosuppressant, and biologics are the three treatment approaches for PACNS. Immunosuppressants are considered the cornerstone to stop the disease progression. Treatment plan is divided into two phases: induction phase and maintenance phase. For induction phase, methylprednisolone and cyclophosphamide are used to achieve remission. This combination seems to have same effectiveness as methylprednisolone alone but has fewer relapses^5^. 1 g/day pulse intravenous methylprednisolone should be ordered for 3–5 days followed by 1 mg/kg/day oral prednisolone. A full dose of oral prednisolone is continued for 4–6 weeks, which is followed by slow tapering over a period of 6 months^11^. For patients not tolerating steroids and immunosuppressants, biologic agents like rituximab and tumour necrosis factor alpha inhibitors could be used as they show the same ability to induce remission compared to glucocorticoids and immunosuppressants. In addition, gastric ulcer and venous thrombosis prophylactic medications should be included in the induction treatment plan^5^.

PACNS maintenance therapy should start 4–6 months after initiation of the induction therapy. This phase helps to prevent future relapses and long-term disabilities. Methotrexate, mycophenolate mofetil, and azathioprine are the disease modifying drugs used during this phase^11^. Pneumocystis pneumonia and gastric ulcer prophylaxis are also implemented during the maintenance treatment plan^5^.

### Prognosis

Morbidity, mortality rate, and relapses have significantly decreased in favour of the above regimen. Patients with this disease has a mortality rate reaching about 6–16%^11^, and relapses happen in approximately one-third of the patients^6^. They may have relapses of existing or new symptoms other than headache, and the existing lesions on MRI may worsen or new lesions may develop^11^. A comprehensive review of 76 biopsy-proven cases by Lu *et al*.^17^ found that a patients with recurrent relapses had a higher incidence of symptomatic epilepsy, a longer time window for biopsy, pathological tissue with more CD20, and a higher percentage of intracranial enhancement.

## Conclusion

Primary central nervous system angiitis is a life-threatening disease that requires accurate diagnosis and treatment. This case highlights the challenges of diagnosis as the disease has diverse clinical features, and there is no laboratory or radiological modality with sufficient sensitivity and specificity. As the treatment involves immunotherapy with its significant side effects, we emphasize the role of histopathology in confirming the diagnosis and excluding other mimickers of vasculitis. In addition, it is possible to prevent the pitfalls of misdiagnosis by being aware of the clinical presentations of mimickers and characteristics that can differentiate PACNS from vasculitis.

## Ethical approval

This study is exempt from ethical approval in our intuition.

## Consent

We obtained verbal and written informed consent from the patient for this case report. A copy of the written consent is available for review by the Editor-in-Chief of this journal on request.

## Source of funding

The study did not receive any financial help.

## Author contribution

Y.N.A.A., H.A.S. Study concept or design: Z.M.M.Z., Y.N.A.A., H.A.S., M.M.A. Writing the manuscript: Z.M.M.Z., Y.N.A.A., H.A.S., M.M.A. Review and editing the manuscript: Z.M.M.Z., Y.N.A.A., H.A.S., M.M.A.

## Conflicts of interest disclosure

The authors have no conflict of interests to declare.

## Guarantor

Mohammad M. Abbas.

## Data availability statement

The authors confirm that the data supporting the findings of this study are available within the article and its Supplementary material.

## Provenance and peer review

Our paper was invited.
